# The Genetic Relatedness in Groups of Joint-Nesting Taiwan Yuhinas: Low Genetic Relatedness with Preferences for Male Kin

**DOI:** 10.1371/journal.pone.0127341

**Published:** 2015-06-18

**Authors:** Mark Liu, Quen-Dian Zhong, Yi-Ru Cheng, Shou-Hsien Li, Shu Fang, Chang-En Pu, Hsiao-Wei Yuan, Sheng-Feng Shen

**Affiliations:** 1 Biodiversity Research Center, Academia Sinica, Taipei, Taiwan; 2 School of Forestry and Resource Conservation, National Taiwan University, Taipei, Taiwan; 3 Department of Life Science, National Taiwan Normal University, Taipei, Taiwan; 4 Scientific and Technical Research Center Investigation Bureau, Ministry of justice, Hsin-Tien, Taiwan; Macquarie University, AUSTRALIA

## Abstract

The relative importance of direct and indirect fitness and, thus, the role of kinship in the evolution of social behavior is much debated. Studying the genetic relatedness of interacting individuals is crucial to improving our understanding of these issues. Here, we used a seven-year data set to study the genetic structure of the Taiwan yuhina (*Yuhina brunneciceps*), a joint-nesting passerine. Ten microsatellite loci were used to investigate the pair-wised relatedness among yuhina breeding group members. We found that the average genetic relatedness between same-sex group members was very low (0.069 for male dyads and 0.016 for female dyads). There was also a low ratio of closely-related kin (r>0.25) in the cooperative breeding groups of yuhinas (21.59% and 9.68% for male and female dyads, respectively). However, the relatedness of male dyads within breeding groups was significantly higher than female dyads. Our results suggest that yuhina cooperation is maintained primarily by direct fitness benefits to individuals; however, kin selection might play a role in partner choice for male yuhinas. Our study also highlights an important, but often neglected, question: Why do animals form non-kin groups, if kin are available? We use biological market theory to propose an explanation for group formation of unrelated Taiwan yuhinas.

## Introduction

Since Hamilton’s seminal work [1,2], the concept of inclusive fitness has played a crucial role in the study of social behavior. Inclusive fitness theory revolutionized thinking about the evolution of social behavior by explaining how selection can act on a gene through its effects on its bearer’s relatives. An individual may enhance its fitness directly through the production of its own offspring, and indirectly through its positive effects on the reproduction of relatives. The influence of kinship on individual behavior has been demonstrated in many studies [3,4,5]. However, the importance of kinship to the evolution of social behavior has been much debated [6–9]. Knowledge of the genetic relatedness between interacting individuals is crucial to measuring direct and indirect fitness and, thus, is crucial to understanding social behavior.

Cooperative breeding occurs when at least one individual, in addition to the breeding male-female pair behaves parentally toward a single brood [10,11]. There are several types of avian cooperative breeding, including helping at the nest by offspring that have delayed dispersal, plural breeding, in which females produce separate nests within a larger social group, and joint nesting, in which more than one individual of one or both sexes contributes genes to the clutch of a single breeding group [10,12]. Although forming large social groups containing more than one breeding pair is a common phenomenon, dispersal patterns and relatedness often differs among joint-nesting and plural breeding species [10,13]. These provide fascinating systems in which to explore some interesting questions, such as: 1) Why do some species cooperate with kin while other species cooperate with non-kin? [14] and 2) What are the benefits of cooperation in non-kin groups [13]?

Improved molecular techniques provide a direct method of investigating kinship. Molecular methods can reveal unexpected relationships among group members [15]. For example, crows immigrating into breeding groups are related to the breeders [16]. Inferring kinship based soley on field observations can be misleading because offspring can be the result of extra-pair fertilizations [15]. In addition, relatedness within social groups may be elevated above that inferred from immediate pedigree links because distant pedigree connections and limited dispersal in previous generations can affect local genetic structure [17]. On the other hand, marker-based estimates of relatedness may also be misleading due to problems such as, low power of the genetic makers [18] or false parentage exclusion [19] Thus, combining wild pedigree data and molecular methods are essential for obtaining an accurate understanding of the genetic structure in groups of social animals.

Here we examine the genetic structure of a cooperative breeding passerine, the Taiwan yuhina (*Yuhina brunneiceps*, referred to as yuhinas hereafter); a joint-nesting species in which 90% of groups breed jointly. Yuhina breeding groups are comprised of one to three socially monogamous pairs, sometimes with one or more unpaired individuals. The members of each cooperative breeding group share the labor of nesting, incubation and provisioning. There is a linear dominance hierarchy among the pairs in each group in which male status is fixed, and females adjust their behavior to match their mates’ status [20,21]. Determining the genetic structure of yuhina breeding groups, in which both males and females contribute genes to multiple clutches, will provide insight into potential evolutionary explanations for their cooperation and enhance our understanding of joint-nesting systems and cooperative breeding behaviors.

The objective of this study is to test whether kin selection is a potential evolutionary explanation for the joint-nesting behavior of yuhinas. Because the problems associated with inbreeding depression, it is rare for group members of both sexes to be comprised of kin in cooperative breeding groups [22,23]. Therefore, if most same-sex dyads in a joint-nesting group are relatives, then indirect benefits could be sufficient for cooperative breeding behavior to be maintained by kin selection. However, if most of the same-sex dyads are not related, then direct fitness of individuals must maintain this breeding behavior. We test the importance of indirect fitness by examining (1) the relatedness of groups of cooperatively breeding yuhinas, (2) whether relatedness varies with sex and (3) whether yuhinas have a greater likelihood of forming kin groups than random expectation. Unlike some cooperatively breeding vertebrates, we did not detect elevated relatedness in the joint-nesting groups of yuhinas. These results indicate that yuhina cooperation is maintained primarily by direct genetic benefits to individuals. We discuss hypotheses that potentially explain reasons yuhinas form groups with unrelated individuals.

## Materials and Methods

### Study site & population

This study was conducted at the National Taiwan University Highlands Experiment Farm at Meifeng, Nantou County, in the mountains of central Taiwan (24°05’N, 121°10’E, elevation 2150 m). A 50 ha area with a system of small roads was chosen wherein the activities of the breeding groups could be monitored. In total, 69 year-groups were studied during seven field seasons (April- September, 1997–2001, 2003–2004). A year-group is defined as the number of groups observed each year over the study period. There were 6, 7, 6, 8, 18, 9 and 15 groups in 1997, 1998, 1999, 2000, 2001, 2003 and 2004, respectively. Additional details of the study site can be found in an earlier publication [24].

The yuhina breeding season extends from March to September, and yuhinas make 5–6 nesting attempts each breeding season[25]. Although a linear hierarchy occurs in each sex, each member of a cooperative breeding group contributes constantly to the nesting attempt and, therefore, the mean reproductive skew index is low (0.19)[25]. All members of a cooperative breeding group share the labor of nesting, incubating and provisioning. After the breeding season, the breeding groups disband and the yuhinas join larger ‘feeding groups’ comprised of more than 20 individuals. Yuhinas remain in feeding groups until the start of the next breeding season, in March, when smaller cooperative breeding groups form [26].

### Behavioral observations

Adult yuhinas were caught using mist nets. Each individual was banded on both legs with one metal ring and a unique combination of three colored plastic rings. Approximately 20–70 ul of blood was taken from the brachial vein of each bird. Blood samples were preserved in Queen’s lysis buffer [27] or 99% alcohol. We monitored the activity of each breeding group every 1–3 days during the breeding season. A cooperative breeding group was defined as a set of individuals showing parental behavior toward the young of a single nest. The membership of each group was monitored, and the position of each bird within the group hierarchy was determined using displacement and chase observations [28,29]. Higher-ranked individuals consistently chase and displace lower-ranked, same-sex members of the group. The linear hierarchy of each sex was determined by the consistent patterns of displacement and chasing [26]. For individual pairs that participated in more than one breeding group in the same year, the paired data are calculated only once to minimize pseudo-replication.

### Sex determination and molecular analysis

Blood samples were taken along with the regular banding procedures. Only sampled individuals with recorded behavioral data were included in the molecular analyses. A total of 171 different individuals were used in the analyses. DNA was extracted from blood samples via the lithium chloride method [30]. We sex-typed individuals with 2550F and 2718R primers [31]. The polymerase chain reaction (PCR) was carried out at 94 for 2 min, with 20 cycles at 94°C for 30 sec, 47.9°C for 60 sec, and 72°C for 30 sec, followed by 72°C for 5 min. PCR products were electrophoresed on a 2% agarose gel at 100V for 30 min and visualized with ethidium bromide under UV light.

Each individual was genotyped at 7–10 microsatellite loci for kinship assignment and relatedness pattern analysis. Primer sequences for GATA 8, GATA11, GATA13, GATA 15 and GATA 22 were obtained from [32]; lsgata07 and lsgata17 and lsgata21 were obtained from [25]; T39 was obtained from [33]; and 1311 was an unpublished primer pair (Li, unpubl. data; Table 1). Polymerase chain reactions were carried out in 10-ul reaction volumes containing 50ng genomic DNA, 0.4U Taq DNA polymerase (Amersham), 0.3M of a TAMAR, FAM or HEX, fluorescently labelled primer, 0.5mM dNTP, 10mM Tris-HCl, pH 9.0, 50 mM KCl, and 1.5mM MgCl_2_. All reactions were carried out as follows: an initial denaturation step at 94°C for 2 min, then 30 cycles of 94°C for 30 sec, 60 sec at the locus-specific annealing temperature of each primer (Table 1), elongation at 72°C for 30 sec, and a final elongation step at 72°C for 3 min. The size of PCR product was determined using a Megabase 500 autosequencer (Amersham Biosciences) using Genetic Profile 2.0 software (Amersham Bioscience).

**Table 1:** Microsatellite loci used to assess relatedness and kin relationships between 171 Taiwan yuhinas (81 males and 86 females, 4 unknown sexes): annealing temperature (*T*_*a*_), number of alleles per locus (*N*_*A*_), observed heterozygosity (*H*_*O*_), results of the Hardy-Weinberg equilibrium test (*H-W*), and proportion of null alleles (*N*_*a*_).

Locus	Primer sequence	*Ta*	Allele size	*NA*	*HO*	*He*	*H-W*	*Na*
1311	Unpublished sequence*	59.3°C	120–190	26	0.87	0.92	NS	0.029
GATA08	Huang et al. 2004	50°C	90–100	2	0.509	0.5	NS	-0.01
GATA11	Huang et al. 2004	60°C	170–180	4	0.383	0.428	NS	0.054
GATA13	Huang et al. 2004	57.5°C	151–171	7	0.64	0.717	NS	0.055
GATA15	Huang et al. 2004	59.3°C	110–150	7	0.759	0.763	NS	0
GATA22	Huang et al. 2004	53.7°C	210–230	7	0.527	0.559	NS	0.025
lsgata 07	Yeung et al. 2004	61.1°C	238–272	14	0.72	0.748	NS	0.016
lsgata 17	Yeung et al. 2004	47.9°C	104–128	7	0.65	0.653	NS	0.003
lsgata21	Yeung et al. 2004	53.6°C	95–111	5	0.544	0.544	NS	-0.011
Titgata39	Wang et al. 2005	52.3°C	214–230	7	0.712	0.736	NS	0.02

*L:AACAAACTGTTTCATTCTCCTCC;R:CTGATGTCATATAACAGTGACAGG

With the prior microsatellite loci screening using 100 individual samples, we detected the expected range of allele size and characteristic peak pattern. To ensure that amplification of alleles is consistent throughout the duration of a study, a positive control was run with every PCR batch [34]. Cervus 3.0.3 [35] was used to test all loci for deviation from Hardy-Weinberg equilibrium and estimated the proportions of null alleles for each locus. GENEPOP 4.1.2 [36] was used to detect linkage disequilibrium between all pairs of loci.

To avoid scoring errors, each sample containing homozygotes was genotyped at least twice to inspect the large allele dropout, and the microsatellite data were scored manually to decrease the error rate because of the stuttering band. Loci with unclear peak information, linkage equilibrium or Hardy-Weinberg disequilibrium were excluded from genotype analysis. We estimated the microsatellite scoring error rate in the dataset by randomly resampling individuals from the population and re-genotyping them, the average re-analyzed rate for all locus was 60%. The original electropherograms were compared to the test electropherograms to evaluate levels of large allele dropout and technical sizing errors. We calculated the error rate per allele and per reaction for each locus and then calculated the average error rate from all loci combined [37]. The fieldwork including field observation, mist netting, banding and blood sampling of birds was approved by the Council of Agriculture of Taiwan (permission no. 092013350-A1), and the field access was approved by the National Taiwan University Highlands Experiment Farm (permission no. 95-3-2_103-2-9). Experiment and animal care procedure was approved by Biological research registration form – Biosafety committee of Academia Sinica (permission no. BSF0410-00002212).

### Kinship assignment and relatedness analysis

One method used to characterize kin structure in each study group was to calculate pairwise relatedness between all adult yuhina pairs from the same year using the Queller & Goodnight’s formulation [38] implemented in SPAGeDi 1.3 [39]. To assess whether our data from 10 loci could be used to estimate of relatedness between individuals of unknown kinship, we calculated the relatedness values of known pedigree relationships in 4 different categories (parent-offspring dyads, full sibling dyads, half sibling dyads and randomized dyads) and compared them to the hypothetical values using one-sample T-Tests. The pedigree relationship data were obtained from Cervus 3.0 parentage assignment tests within each breeding group. The theoretical values of relatedness were 0.5, 0.5, 0.25 and 0.0 for parent-offspring pairs, full-sibling pairs, half-sibling pairs and all individual pairs in the Meifeng population, respectively. We use this method to determine whether relatedness values for a given subset of individuals (all individuals with group members of the same sex) were different from the expected level of genetic similarity of all the individuals successfully sampled at the study site. In addition, we also performed rarefaction analysis to decide the number of loci necessary to acquire consistent relatedness using program RERAT[40].

We examined whether relatedness of cooperative dyads of same sex was different from randomly sampled dyads from population using randomization test with Rstudio software [41]. The null distribution of t statistics was generated by 1000 cycles of resampling from dyads in population. The difference between the means of cooperative dyads and the population was then tested by comparing the observed t value with the null distribution.

Another method for characterizing kin structure examines the prevalence of closely related individuals within focal cooperative breeding groups and the study population. For each pair of individuals, we used KINGROUP [42] to compare the relative likelihoods the two birds were closely related (r>0.25) or unrelated (r<0.25). This software uses the likelihood formulas proposed by [43]. Given a specific hypothesis about particular pedigree relationships, KINGROUP tested the hypothesis that alleles were identical by descent (IBD) as a consequence of the primary hypothesis (kin relationship, r>0.25) or null hypothesis (e.g. unrelated kin relationship, r<0.25). Hypotheses were accepted or rejected on the basis of the log-likelihood ratio between the two hypotheses (primary/null). The pedigree relationships specified by the primary and null hypotheses were confirmed with a 5000 random pair sampling simulation. The data were used to accept or reject the null hypotheses with 95% confidence. The proportions of closely-related kin relationships of each sex in cooperative breeding groups were compared with the chi-square test.

### Sex-biased relatedness patterns at the breeding group and population levels

Mean relatedness between birds of the same sex was estimated at the cooperative breeding group level (relatedness between co-breeders of the same sex) and at the population level (relatedness between all males and relatedness between all females in the study population). If there is sex-biased dispersal, mean relatedness between the individuals of the dispersing sex should be lower than the mean relatedness between individuals of the more philopatric sex. If there is no bias in dispersal or there is bisexual dispersal, no significant differences between sexes are expected in the mean relatedness of individuals at the cooperative breeding group level or at the population level. For dyads within the same breeding group, we analyzed data based on two assumptions, whether dyads choice among years was independent or dependent. Statistical tests were run in RStudio software (Version 0.96.330)[41] and all distributions were tested for normality before t-tests were performed. Relatedness between individuals of the same sex at the population level is not normally distributed, so mean relatedness between all males and between all females in the Meifeng population was compared with the Mann-Whitney U-test.

## Results

### Genetic analyses

A total 171 individuals were genotyped at 7–10 loci. 91% of the population (n = 155) was genotyped successfully in either 9 or 10 loci. Most of the loci were polymorphic with 2–26 alleles per locus and the observed Ho in this population ranging from 0.38 to 0.87. The combined-over-loci genotypic error rate was 0.026 per reaction and 0.016 per allele and was caused by mistakes in allelic dropout or gel scoring, rather than false alleles. Exact tests of Hardy Hardy-Weinberg equilibrium cannot reject the null hypothesis of random mating for all loci after Bonferroni correction, and the proportions of null alleles between each locus ranged from 0 to 0.055. Tests for linkage disequilibrium showed that all loci were non-significant after Bonferroni correction and showed no significant deviation from Hardy-Weinberg equilibrium (Table 1).

### Relatedness of known pedigree relationships

**Fig 1:**
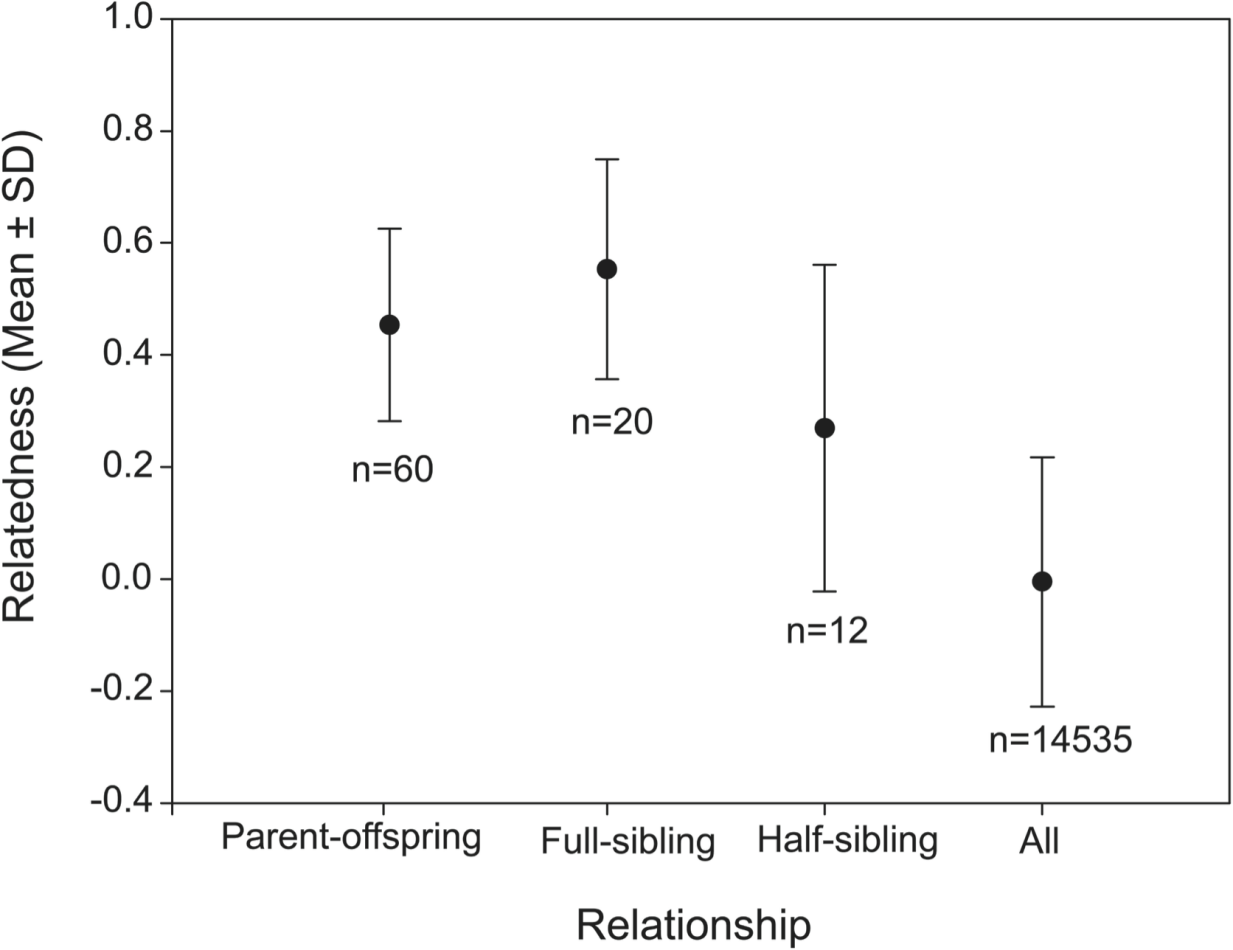
The mean relatedness (±SD) of Taiwan yuhinas in each type of pedigree relationship. The mean relatedness of individuals in each relationship type did not differ significantly from the hypothetical values (Parent-offspring = 0.5, Full siblings = 0.5, Half siblings = 0.25 Randomized pair = 0), except that the estimate of parent-offspring relatedness is slightly lower.

**Fig 2:**
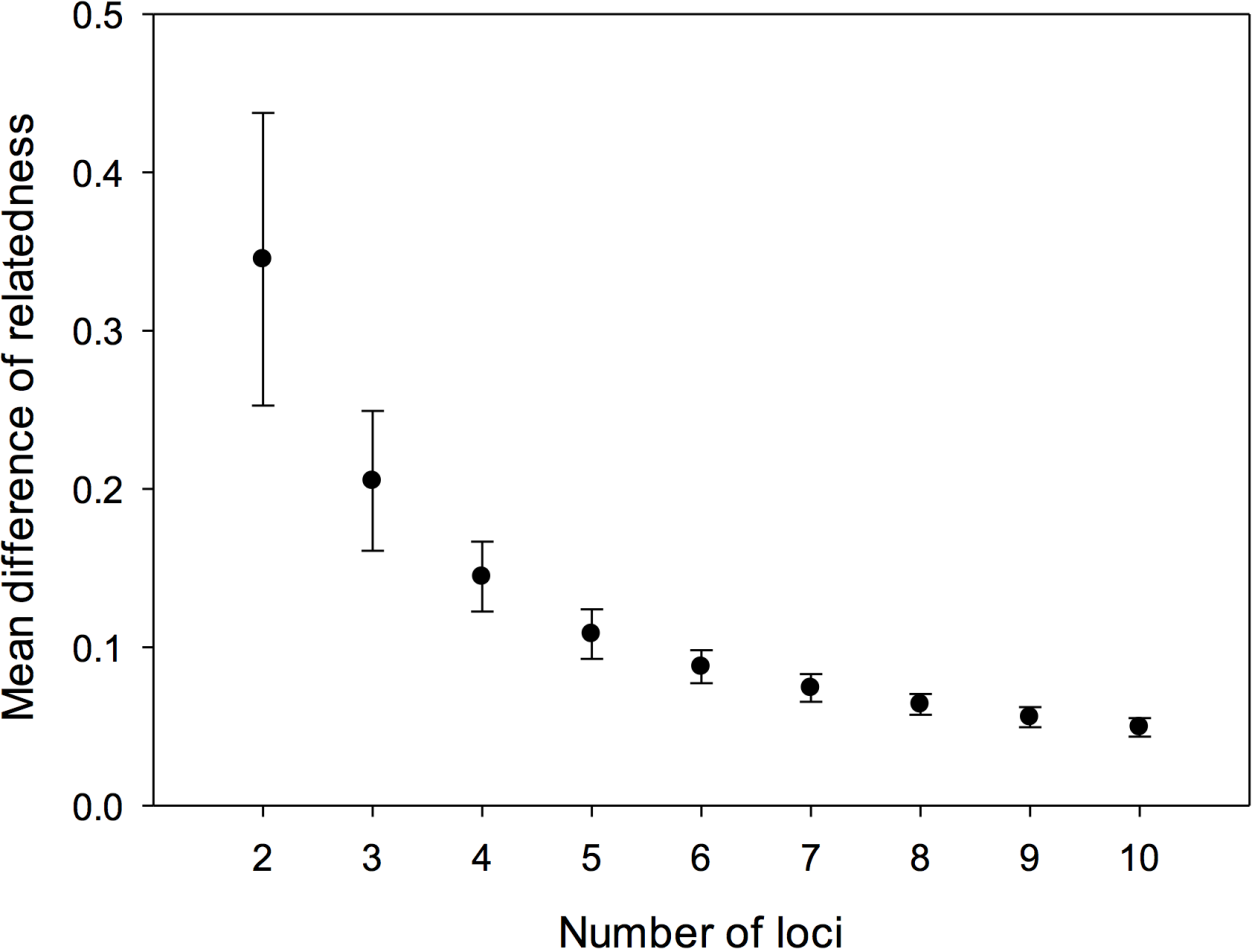
Rarefaction analysis on difference of relatedness estimates when adding additional locus in yuhinas. Mean difference and standard deviation of relatedness estimates were derived from 1,000 simulations using RERAT online software.

To determine whether data from the 7–10 loci could be used to estimate the relatedness between individuals of unknown relatedness, we compared the mean relatedness values of each known pedigree relationship to the hypothetical relatedness values in 4 categories: Parent-offspring dyads, r = 0.5; Full-sibling dyads, r = 0.5; Half-sibling dyads, r = 0.25 and randomized dyads, r = 0). The known pedigree relationship data were obtained from Cervus 3.0 parentage assignment tests within each breeding family. The Cervus settings included an 1% error rate of genotyping and 95% confidence intervals were used as the cut off point. Pairs identified as siblings were members of a single nest from a single pair of genetic parents. Similarly, pairs identified as half siblings were determined to have a shared mother but have different fathers, or vice versa. A total of 36 families were used to assign kinship relationships into 3 different categories: Parent-offspring dyads (n = 60), Full-sibling dyads (n = 20) and Half-sibling dyads (n = 12). Relatedness values obtained from the microsatellite data well represented each kinship categories where the one-sample T-tests shows R-values of parent- offspring (0.453 ± 0.022, n = 60, t = -2.09, df = 59, p = 0.04), full-sibling pairs (0.553 ± 0.044, n = 20, t = 1.212, p = 0.24), and half-sibling pairs (0.269 ± 0.078, n = 12, t = 0.22, p = 0.83) did not differ significantly from the coefficients of relatedness expected from the hypothetical values (except the parent-offspring category had slightly lower r values (r = 0.453) compared to the hypothetical value (r = 0.5) (Fig 1). The mean relatedness of all individuals in the Meifeng population did not deviate from an outbreed population (-0.006 ± 0.003, n = 5545, t = 1.93, p = 0.05). These results demonstrate that the microsatellite loci and estimator that we used to estimate relatedness accurately characterized the relatedness of individuals with a known pedigree. The rarefaction analysis revealed that there was a ≤ 0.07 average pairwise difference in relatedness value calculated using 7 loci, and ≤ 0.05 when using 10 loci (Fig 2), indicating that use of 10 loci provided sufficient relatedness estimates.

### Relatedness of breeding groups compared to random expectations in the population

Relatedness within breeding groups differed with sex. Relatedness between males in the same cooperative breeding groups was significantly different from the random expectation of relatedness between all males in the Meifeng population (randomization test, p = 0.013), although the average observed relatedness was far from kin (r_mm_ = 0.069) (Table 2). Relatedness of female dyads in the same cooperative breeding group was not significantly different from the random expectation of relatedness among all females in the Meifeng population (randomization test, p = 0.712)(Table 2). The average observed relatedness among females also indicated non-kin cooperation (r_ff_ = 0.016)(Table 2). Moreover, male dyads with higher relatedness were significantly more likely to cooperate in multiple years suggesting a tendency for related males to continue cooperation across years, but this relationship was not found among the female dyads (interaction between sex and cooperative repeat: F_1,3425_ = 5.48, P = 0.02; male dyads: slope = 0.057, P < 0.001; female dyads: P = 0.90).

**Table 2:** Comparison of relatedness of male dyads and female dyads in breeding groups with random dyads in the population using permutation randomization tests.

		Male-male dyads		Female-female dyads	
		population	breeding group	population	breeding group
Dependent among years	N	1311	88	1428	93
	Mean ± SE	0.003±0.006	0.069±0.027	-0.008±0.006	0.016±0.021
	p value	-	0.013	-	0.712
Independent among years	N	1870	113	1913	115
	Mean ± SE	0.008±0.005	0.093±0.025	-0.003±0.005	0.001±0.02
	p value	-	0.002	-	0.812

Analyses were performed under two different assumptions: dyad choice among years was dependent (same pair was counted only once) or independent among years.

**Fig 3:**
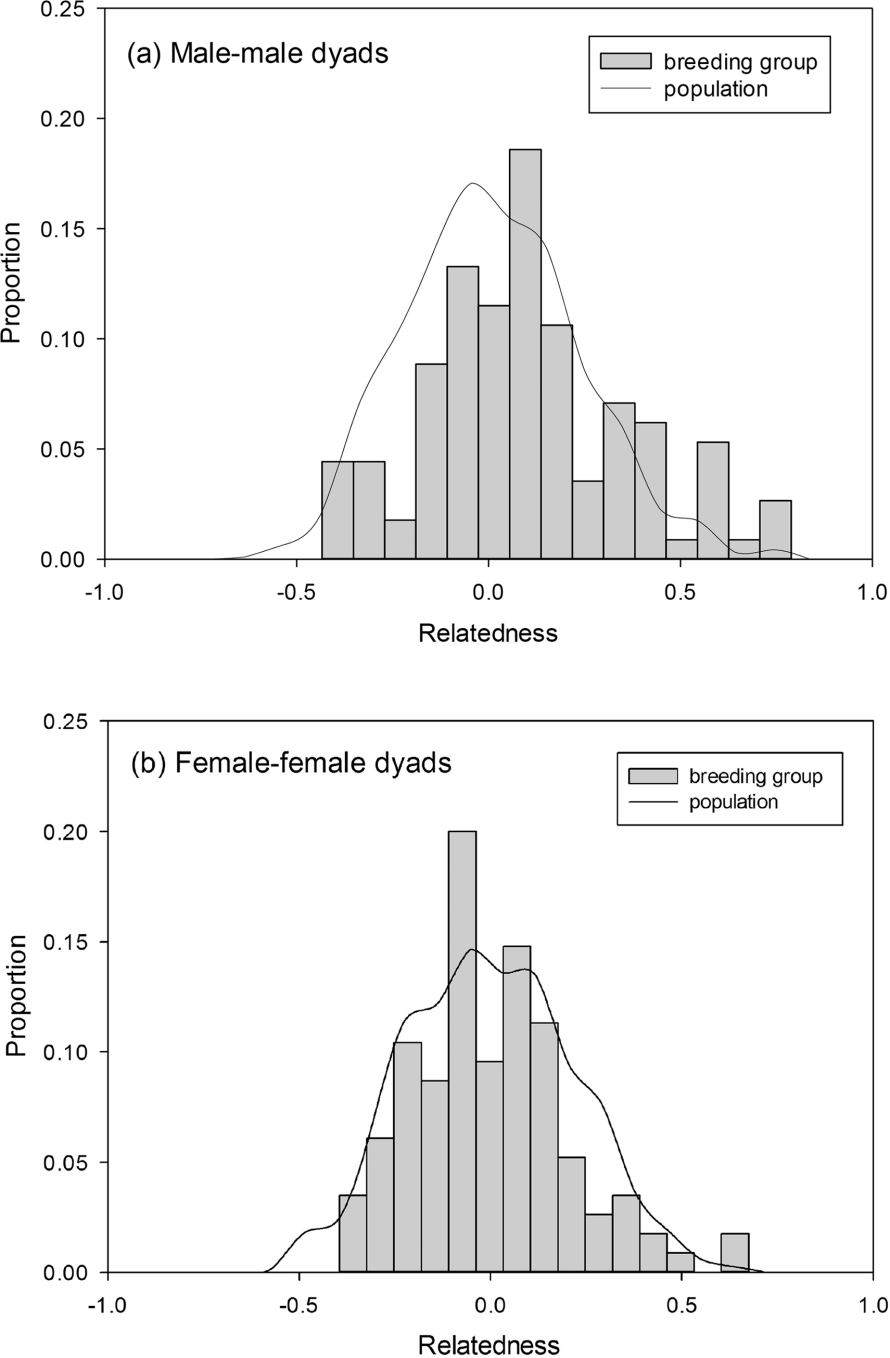
The distribution of relatedness among same sex, co-breeding individuals in the same group and among all same-sex dyads of yuhinas in the Meifeng population. There was no significant difference between the mean relatedness of co-breeders and all individuals in the population. (a) pairwise relatedness distribution among male-male dyads in the same group and among all male-male dyads in the population; (b) pairwise relatedness distribution among female-female dyads in the same group and among all female-female dyads in the population.

The results from plotting the observed male dyads relatedness distribution against the relatedness distribution from two randomly selected males in the population show that the distribution of observed male-dyad relatedness is similar to the population curve, yet there were slightly more closely-related dyads than would be expected by chance (Kolmogorov-Smirnov test, P = 0.049). The observed relatedness distribution of female dyads, on the other hand, matched the population curve (Kolmogorov-Smirnov test, P = 0.287). Both observed relatedness distributions formed a symmetrical bell shape and centred on the 0 degree of the relatedness (Fig 3).

The kin relationships classified by the program KINGROUP exhibited a low ratio of closely-related kin in the cooperative breeding groups. Only 29 dyads of male co-breeders (25.7%, n = 113) and 11 dyads of female co-breeders (9.6%, n = 115) were closely related (Table 3).

**Table 3:** The proportion of closely related kin in cooperative breeding groups of Taiwan yuhinas at Meifeng (MM: male-male pairs; FF: female-female pairs; MF: male-female pairs).

	Breeding group	Pairs	MM pairs	FF pairs	MF pairs
Sample size	69	564	113	115	336
Number of kin group or dyads	38	69	29	11	29
Percent (%)	55.07	12.23	25.66	9.57	8.63

### Relatedness of dyads within cooperative breeding groups

Within cooperative breeding groups, the relatedness of males in the same group was significantly different from relatedness of females in the same group (Table 2; Mann-Whitney test: U = 4998, p = 0.01). In addition, the proportion of closely-related male dyads was significantly higher than the proportion of closely-related female dyads (Table 3; χ^2^ = 9.13, p = 0.003).

### Relatedness between males and females in the population

At the population level, males were not more closely related to one another than were females (Table 2; Mann-Whitney test: U = 960614, p = 0.235).

## Discussion

We show that the average relatedness between co-breeding Taiwan yuhinas of both sexes is relatively low, indicating that yuhina breeding groups are comprised mainly of non-relatives. Therefore, increases in indirect fitness arising from cooperative breeding should be minimal, and inclusive fitness is not crucial factor promoting the evolution of cooperation in yuhinas. Nonetheless, 21.59% of male dyads and 9.68% of female dyads were found to be closely related, suggesting that kinship still influences partner choice in yuhinas.

### Low genetic relatedness in cooperative breeding groups of yuhinas

Unlike most species of cooperatively breeding birds, yuhinas breed in groups comprised mainly of unrelated individuals. When cooperating with unrelated individuals, group members face higher risks of “cheating”, in which group members deceive each other, and “free-riding”, in which individuals receive benefits without offering the commodity provided by other individuals [44]. Why animals form non-kin groups is an important but often neglected question, presumably because most previous studies in cooperative breeding have focused on studying delayed dispersal in offspring. Direct fitness of cooperative breeding is expected to explain the maintenance of non-kin groups. However, individuals should still prefer to form groups with kin because their genetic interests are more aligned. Using the biological market concept [5,44,45], Reeve [46] applied evolutionary game theory to model the joint evolution of reproductive partitioning and the genetic composition of cooperative breeding groups. Reeve proposed two different models to explain the formation of non-kin groups: (1) the “bidding game”, which considers the effect of partner choice from the subordinate’s point of view, given that some dominants in the population cannot recruit enough group members [see also 44]; and (2) the “beggars-can’t-be-choosers game”, which examines what happens to dominants who prefer to cooperate with kin, when they accept or reject an unrelated joiner and no relatives join the group [14].

In the bidding game model, there are not enough subordinates in the population to meet the demands of every dominant individual, and the cost to subordinates of sampling multiple dominants is little or none. In this scenario, dominants compete for help and each should provide a reproductive share “bid” to subordinates. Depending on the market, subordinates could reap most of the benefits of cooperation [5]. Unrelated partners are accepted because of the direct fitness benefits of cooperation. Also, given the lack of subordinates in the population, there is no inclusive fitness advantage for dominants to prefer related partners because the subordinates can always join other groups and breed. In the second model, although dominants prefer to cooperate with kin, non-relatives are accepted because the risk is high of not encountering a related joiner. Both models predict that non-relatives are more likely to form groups when ecological constraints are strong and/or benefits of grouping—the direct fitness of cooperation—are high.

In yuhinas, most (90%) breeding groups are cooperative, and the group-size effect on labor division and adult survival rate indicates the benefits of joint nesting are large [47]. These results are consistent with the assumptions of Reeve’s models [46]. In addition, because most yuhinas breed with non-relatives and the majority of offspring disperse after fledging, it appears that most yuhinas disperse to join other groups. The small, overlapping home ranges (2–7 ha) of yuhinas should also allow individuals to sample different groups easily [24]. This makes the partner choice (or bidding) process very likely in yuhinas. Presumably, the sampling cost is small in yuhinas. The beggars-can’t-be-choosers idea also seems to play a role in yuhina group formation. Yuhinas have a long (5–6 months) breeding season, and multiple-nesting attempts (up to nine each season) is the norm [26]. However, because of harsh, unpredictable weather and predation, only about 22% of nests are successful [26]. Adults usually chase fledglings away about two weeks after fledging, unless it is the last nesting attempt of the season (S.-F. Shen, unpubl. data). Therefore, this may reduce the likelihood of recruiting kin. In addition, because male-male dyads are more closely related than female-female dyads suggesting kin selection might still play a role in the partner choice among males in yuhinas. This result supports the beggars-can’t-be-choosers game, in which kin selection plays a role acceptance of group members but also argues that kin selection is not the key reason for group formation (S.-F. Shen, unpubl. data). However, because both models are based on the complete control assumption, the role of intra-group conflict is neglected. To better understand the formation of non-kin groups, more biologically realistic models are needed; models need to incorporate intra-group conflict, the mechanisms of partner choice, and the conflict between the current group members and potential joiners [48].

The relatedness patterns of joint-nesting and plural breeding birds are probably the most varied among animal societies [13]. In a recent review, Riehl [49] shows 31 of 54 cooperative polygamous species were comprised of unrelated co-breeders. A review by Koenig et al. [13]demonstrates that, in 15 bird species, relatedness between co-breeding females is low in 7 species, moderate in 2 species (50% and 62% of pairs were related) and high in 6 species. In males, relatedness between co-breeders is low in 5 species, moderate in 2 species (23% and 50% were related) and high in 8 species. Breeding groups of different joint-nesting and plural breeding species can be formed by delayed-dispersal of offspring, staying in the natal group, or by immigration of unrelated individuals. In Acorn woodpeckers (*Melanerpes formicivorus*) [50,51], females and males are usually related to the same-sex co-breeders. Female woodpeckers usually disperse with relatives to compete for vacant territory. In contrast, in yuhinas, Groove-billed anis (*Crotophaga sulcirostris*) [52,53], Guira cuckoos (*Guira guira*) [54,55] and Greater anis (*Crotophaga major*)[56], group members are not related to same-sex co-breeders. In many of the studies described above, relatedness is inferred from dispersal patterns, or only first-order relatives can be determined due to small sample size or the low resolution of genetic markers, but see [51,57]. Clearly, more studies on the genetic structure of join-nesting and plural breeding species are needed before we can understand the factors leading to the evolution of these breeding systems.

### Female-biased dispersal

The dispersal of individuals is one of the key factors affecting the genetic structure of populations [58–60]. If genetic similarity is greater for one sex, it suggests that movement by individuals of this sex is more limited than it is for individuals of the opposite sex [61–63]. Because male yuhinas are more closely related to other male co-breeders than to females, dispersal is probably female-biased. In agreement with the genetic data, banding data from this population shows that male offspring are more likely to stay in their natal groups after fledging than are females (S.-F. Shen, unpubl. Data). Female-biased dispersal might also play a role in the kin structure in yuhina’s breeding groups. Further investigation into the recruitment process of group members is warranted to gain insight into non-kin cooperation [49].

### Direct benefits of cooperative breeding

Because within-group relatedness in yuhinas is low, kin selection is unlikely to have played an important role in the evolution of joint nesting in this species. Direct benefits should be the main factor promoting joint-nesting behavior in yuhinas. A previous study found that the per capita clutch size and nesting success did not increase with group size and that nesting success was low and variable both within and between seasons [47]. However, individuals, especially the alpha pairs, living in larger groups reduced their parental workload, and the survival probability of all group members, except alpha males, increased with group size [47]. Larger groups also re-nested faster after nest failure. Thus, joint nesting in yuhinas could be part of a “bet-hedging” strategy to cope with a highly variable environment, as suggested in recent comparative studies of cooperative breeding birds [64–66]. Individuals invest less in each nest attempt, but are able to re-nest faster and make more attempts each year [20]. Some empirical studies suggest that the evolution of cooperation is supported by the benefits of group living [67–69] and recent research suggests that cooperation can evolve in the absence of kin selection [6,70,71]. In Taiwan yuhinas, the high proportion of groups that breed cooperatively and the low genetic relatedness within these groups indicates that cooperative breeding is maintained by strong selection on the direct benefits of these behaviors.

## Conclusions

We show that molecular methods can provide invaluable information and insights about the genetic structure of populations and the relatedness of individuals, which would be difficult or impossible to obtain using only field observations. The yuhina joint-nesting system also provides an excellent opportunity to examine conflicts between co-breeders and the formation of non-kin cooperative groups. Studying non-kin cooperation can help us understand the role of direct fitness in the evolution of cooperative behaviors.
